# (*E*)-1-(4-Meth­oxy­phen­yl)-3-(3,4,5-trimeth­oxy­phen­yl)prop-2-en-1-one

**DOI:** 10.1107/S1600536811028984

**Published:** 2011-07-23

**Authors:** P. S. Carvalho-Jr, L. O. Sallum, A. F. Cidade, G. L. B. Aquino, H. B. Napolitano

**Affiliations:** a Department of Chemistry, State University of Goias, Anapolis, Brazil

## Abstract

The title compound, C_19_H_20_O_5_, was synthesized by reaction of 4-meth­oxy­acetophenone and 3,4,5-trimeth­oxy-benzaldehyde. The aromatic rings form a dihedral angle of 36.39 (7)°. Two intramolecular C—H⋯O hydrogen bonds occur. The crystal packing features weak C—H⋯O inter­actions.

## Related literature

For background to chalcones and the biological activity and derivatives, see: Dhar (1981 ▶); Dimmock *et al.* (1999 ▶). For their applications as organic non-linear optical materials, see: Sarojini *et al.* (2006 ▶) and for their choleretic and hepatoprotective activity, see: Ni *et al.* (2004 ▶). For the synthesis of chalcones, see: Patil *et al.* (2009 ▶). For the potential use of these compounds or chalcone-rich plant extracts as drugs or food preservatives, see: Di Carlo *et al.* (1999 ▶). For related structures, see: Sathiya Moorthi *et al.* (2005 ▶); Cai *et al.* (2011 ▶); Vijay Kumar *et al.* (2011 ▶); Bibila Mayaya Bisseyou *et al.* (2007 ▶). The title compound wss prepared by an aldol Claisen–Schmidt condensation reaction, see: Bandgar *et al.* (2009 ▶, 2010 ▶); Hathaway (1987 ▶).
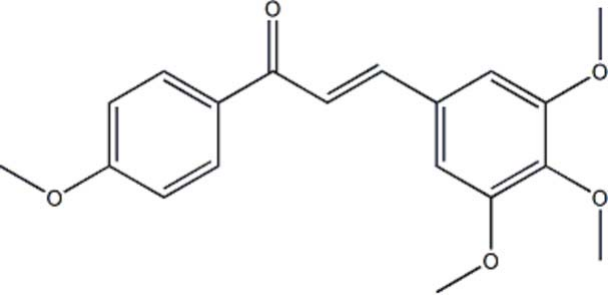


## Experimental

### 

#### Crystal data

C_19_H_20_O_5_
*M* *_r_* = 328.35
Monoclinic, 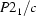

*a* = 7.5770 (1) Å
*b* = 16.2530 (3) Å
*c* = 14.0850 (3) Å
β = 107.528 (1)°
*V* = 1654.02 (5) Å^3^
*Z* = 4
Mo *K*α radiation
μ = 0.10 mm^−1^
*T* = 293 K
0.2 × 0.1 × 0.1 mm

#### Data collection

Nonius KappaCCD diffractometer
27371 measured reflections
3733 independent reflections
2907 reflections with *I* > 2σ(*I*)
*R*_int_ = 0.130

#### Refinement

*R*[*F* ^2^ > 2σ(*F* ^2^)] = 0.056
*wR*(*F* ^2^) = 0.174
*S* = 1.02
3733 reflections
222 parameters
H-atom parameters constrained
Δρ_max_ = 0.27 e Å^−3^
Δρ_min_ = −0.27 e Å^−3^

### 

Data collection: *COLLECT* (Hooft, 1998 ▶); cell refinement: *SCALEPACK* (Otwinowski & Minor, 1997 ▶); data reduction: *DENZO* (Otwinowski & Minor, 1997 ▶) and *SCALEPACK*; program(s) used to solve structure: *SHELXS97* (Sheldrick, 2008 ▶); program(s) used to refine structure: *SHELXL97* (Sheldrick, 2008 ▶); molecular graphics: *ORTEP-3 for Windows* (Farrugia, 1997 ▶); software used to prepare material for publication: *WinGX* (Farrugia, 1999 ▶).

## Supplementary Material

Crystal structure: contains datablock(s) global, I. DOI: 10.1107/S1600536811028984/zj2014sup1.cif

Structure factors: contains datablock(s) I. DOI: 10.1107/S1600536811028984/zj2014Isup2.hkl

Supplementary material file. DOI: 10.1107/S1600536811028984/zj2014Isup3.cml

Additional supplementary materials:  crystallographic information; 3D view; checkCIF report

## Figures and Tables

**Table 1:** Hydrogen-bond geometry (Å, °)

*D*—H⋯*A*	*D*—H	H⋯*A*	*D*⋯*A*	*D*—H⋯*A*
C9—H9⋯O2	0.93	2.51	3.415 (2)	165
C16—H16*A*⋯O2	0.96	2.55	3.484 (2)	165
C18—H18*C*⋯O1^i^	0.96	2.53	3.332 (2)	142

Symmetry code: (i) 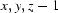.

**Fig. 1.**
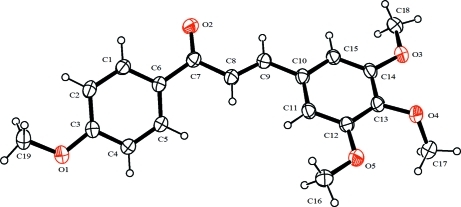
Molecular structre showing 30% probability displacement ellipsoids.

**Fig. 2.**
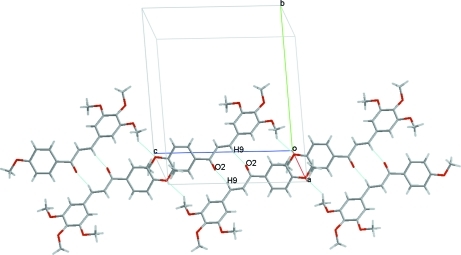
The packing viewed along c axis with C—H···O interactions, indicating the dimer

## supplementary crystallographic information

### Comment

Chalcones are a medicinally important class of compounds and are known for possessing various biological activities such as antibacterial, antiviral, anthelmintic, amoebicidal, antiulcer, insecticidal, antiprotozoal, anticancer, cytotoxic, immunosuppressive activities and other bioactivities (Dhar, 1981; Dimmock *et al.*, 1999). Recently, some chalcones were approved for therapeutical use, such as methoxychalcone (*E*)-3-(4-methoxyphenyl)-1-(2,4-methoxyphenyl)prop-2-en-1-one, marketed in France and Italy, with choleretic and hepatoprotective activities (Ni *et al.*, 2004). Moreover, a literature survey showed dimethoxy and trimethoxychalcone derivatives as effective anti-inflammatory agents (Bandgar *et al.*, 2010).

The (*E*)-3-(3,4,5-trimethoxyphenyl)-1-(4-methoxyphenyl)prop-2-en-1-one is a methoxychalcone which the structure shows two aromatic rings linked by prop-2-en-1-one group. The refined molecular structure is shown in Fig. 1. Due to p-π conjugation, the C*_sp_*^2^—O bonds [O2—C7 = 1.2249 (2) Å] are significantly shorter than the C*_sp_*^3^—O bonds [O1—C19 = 1.4240 (2) Å; O3—C18 = 1.415 (2) Å; O4—C17 = 1.413 (2) Å and O5—C16 = 1.4294 (2) Å]. The methoxy substituted groups around the benzene rings are almost planar and the dihedral angles C5—C6—C7—C8, C6—C7—C8—C9, C7—C8—C9—C10 and C8—C9—C10—C11 are -23.0 (2)°, 169.3 (2)°, 177.7 (2)° and -4.4 (2)°, respectively, indicating the molecule has a non-planar conformation.

The crystal structure is stabilized by C—H···O contacts (Table 1). There is intermolecular hydrogen bonding involving C9 acting as H-bond donor, via H9, to O2 in the adjacent molecules at -x+1, -y+1, -z resulting in a dimer.

### Experimental

The title compound, C_19_H_20_O_5_, has been prepared by the aldol Claisen-Schmidt condensation (Hathaway, 1987; Bandgar *et al.*, 2009) by the reaction of a mixture of 4-methoxy-acetophenone (0,3 mg; 2 mmol) and 3,4,5-trimethoxy-benzaldehyde (0,39 mg; 2 mmol) and NaOH (50% p/v) at 257 K for 24 h. The light yellow solid (m.p. 404.25 - 405.65 K) thus obtained was filtered, washed with water and dried. Crystals of suitable quality for single crystal X-ray diffraction were grown in methanol.

### Refinement

The space group *P*2_1_/*c* was uniquely assigned from the systematic absences. All the H-atoms were placed in calculated positions and treated as riding atoms [C_aro_—H = 0.93 Å and C*sp*^3^—H = 0.96 Å), with a displacement parameter *U*_iso_ set equal to 1.2 times *U*_eq_ that of the parent atom, and C*_sp_*^3^ and aromatic H.

### Crystal data

**Table:**

C_19_H_20_O_5_	*F*(000) = 696
*M**_r_* = 328.35	*D*_x_ = 1.319 Mg m^−^^3^
Monoclinic, *P*2_1_/*c*	Mo *K*α radiation, λ = 0.71073 Å
*a* = 7.5770 (1) Å	Cell parameters from 14574 reflections
*b* = 16.2530 (3) Å	θ = 2.6–27.5°
*c* = 14.0850 (3) Å	µ = 0.10 mm^−^^1^
β = 107.528 (1)°	*T* = 293 K
*V* = 1654.02 (5) Å^3^	Prism, pale yellow
*Z* = 4	0.2 × 0.1 × 0.1 mm

### Data collection

**Table:**

Nonius KappaCCD diffractometer	2907 reflections with *I* > 2σ(*I*)
graphite	*R*_int_ = 0.130
Detector resolution: 9 pixels mm^-1^	θ_max_ = 27.5°, θ_min_ = 2.8°
CCD scans	*h* = −9→8
27371 measured reflections	*k* = −20→21
3733 independent reflections	*l* = −18→17

### Refinement

**Table:**

Refinement on *F*^2^	Secondary atom site location: difference Fourier map
Least-squares matrix: full	Hydrogen site location: inferred from neighbouring sites
*R*[*F*^2^ > 2σ(*F*^2^)] = 0.056	H-atom parameters constrained
*wR*(*F*^2^) = 0.174	*w* = 1/[σ^2^(*F*_o_^2^) + (0.115*P*)^2^ + 0.1311*P*] where *P* = (*F*_o_^2^ + 2*F*_c_^2^)/3
*S* = 1.02	(Δ/σ)_max_ = 0.002
3733 reflections	Δρ_max_ = 0.27 e Å^−^^3^
222 parameters	Δρ_min_ = −0.27 e Å^−^^3^
0 restraints	Extinction correction: *SHELXL*
Primary atom site location: structure-invariant direct methods	Extinction coefficient: 0.085 (10)

### Special details

**Table:**

Geometry. All s.u.'s (except the s.u. in the dihedral angle between two l.s. planes) are estimated using the full covariance matrix. The cell s.u.'s are taken into account individually in the estimation of s.u.'s in distances, angles and torsion angles; correlations between s.u.'s in cell parameters are only used when they are defined by crystal symmetry. An approximate (isotropic) treatment of cell s.u.'s is used for estimating s.u.'s involving l.s. planes.
Refinement. Refinement of *F*^2^ against ALL reflections. The weighted *R*-factor *wR* and goodness of fit *S* are based on *F*^2^, conventional *R*-factors *R* are based on *F*, with *F* set to zero for negative *F*^2^. The threshold expression of *F*^2^ > 2σ(*F*^2^) is used only for calculating *R*-factors(gt) *etc*. and is not relevant to the choice of reflections for refinement. *R*-factors based on *F*^2^ are statistically about twice as large as those based on *F*, and *R*- factors based on ALL data will be even larger.

### Fractional atomic coordinates and isotropic or equivalent isotropic displacement parameters (Å^2^)

**Table:**

	*x*	*y*	*z*	*U*_iso_*/*U*_eq_	
C6	0.28843 (19)	0.01968 (8)	0.72913 (10)	0.0440 (3)	
C3	0.2444 (2)	0.01579 (9)	0.91868 (10)	0.0478 (4)	
O5	−0.33915 (16)	0.26263 (7)	0.32667 (8)	0.0592 (3)	
C7	0.3073 (2)	0.01831 (8)	0.62695 (11)	0.0465 (3)	
O1	0.21258 (17)	0.01994 (7)	1.00875 (8)	0.0613 (3)	
O3	0.06505 (17)	0.22341 (7)	0.13696 (8)	0.0594 (3)	
C13	−0.1306 (2)	0.24141 (9)	0.23514 (10)	0.0496 (4)	
O2	0.41266 (17)	−0.03016 (7)	0.60508 (9)	0.0623 (3)	
C14	0.0304 (2)	0.20629 (8)	0.22489 (9)	0.0472 (3)	
C11	−0.0614 (2)	0.18197 (9)	0.39957 (10)	0.0478 (3)	
H11	−0.0896	0.1757	0.459	0.057*	
C1	0.3390 (2)	−0.04924 (8)	0.78977 (11)	0.0475 (3)	
H1	0.3901	−0.0943	0.7669	0.057*	
C9	0.21256 (19)	0.09013 (9)	0.46478 (10)	0.0464 (3)	
H9	0.3105	0.0638	0.4504	0.056*	
C15	0.1421 (2)	0.15689 (9)	0.29994 (10)	0.0471 (3)	
H15	0.2474	0.1324	0.2917	0.057*	
C10	0.09579 (19)	0.14423 (8)	0.38773 (9)	0.0451 (3)	
C5	0.2205 (2)	0.08797 (9)	0.76692 (11)	0.0501 (4)	
H5	0.1881	0.1351	0.728	0.06*	
O4	−0.24822 (19)	0.28028 (7)	0.15454 (8)	0.0700 (4)	
C12	−0.1763 (2)	0.22889 (9)	0.32326 (10)	0.0475 (4)	
C4	0.2007 (2)	0.08672 (9)	0.86115 (11)	0.0528 (4)	
H4	0.1583	0.1332	0.8861	0.063*	
C2	0.3152 (2)	−0.05237 (9)	0.88338 (10)	0.0502 (4)	
H2	0.3463	−0.0996	0.9221	0.06*	
C8	0.1902 (2)	0.07561 (9)	0.55307 (10)	0.0503 (4)	
H8	0.0955	0.103	0.5694	0.06*	
C17	−0.2865 (3)	0.36460 (11)	0.16310 (12)	0.0693 (5)	
H17A	−0.3621	0.3708	0.2064	0.104*	
H17B	−0.3508	0.3863	0.0985	0.104*	
H17C	−0.1724	0.394	0.1902	0.104*	
C16	−0.3984 (2)	0.24404 (11)	0.41148 (12)	0.0602 (4)	
H16A	−0.4186	0.1859	0.4141	0.09*	
H16B	−0.5116	0.2728	0.4064	0.09*	
H16C	−0.3049	0.261	0.4709	0.09*	
C18	0.2381 (3)	0.19903 (12)	0.12726 (13)	0.0702 (5)	
H18A	0.3358	0.22	0.1825	0.105*	
H18B	0.2513	0.2205	0.0663	0.105*	
H18C	0.2446	0.1401	0.1265	0.105*	
C19	0.2388 (3)	−0.05277 (12)	1.06778 (12)	0.0666 (5)	
H19A	0.1621	−0.096	1.0306	0.1*	
H19B	0.2058	−0.0421	1.1273	0.1*	
H19C	0.3663	−0.0692	1.0852	0.1*	

### Atomic displacement parameters (Å^2^)

**Table:**

	*U*^11^	*U*^22^	*U*^33^	*U*^12^	*U*^13^	*U*^23^
C6	0.0425 (7)	0.0436 (7)	0.0430 (7)	−0.0002 (5)	0.0086 (5)	0.0042 (5)
C3	0.0491 (8)	0.0517 (8)	0.0401 (7)	−0.0033 (6)	0.0098 (6)	0.0043 (5)
O5	0.0536 (7)	0.0738 (7)	0.0466 (6)	0.0147 (5)	0.0095 (5)	0.0039 (5)
C7	0.0463 (7)	0.0433 (7)	0.0502 (8)	0.0004 (6)	0.0150 (6)	0.0046 (5)
O1	0.0772 (8)	0.0628 (7)	0.0459 (6)	0.0058 (5)	0.0215 (5)	0.0096 (4)
O3	0.0722 (8)	0.0672 (7)	0.0393 (5)	0.0078 (5)	0.0175 (5)	0.0058 (4)
C13	0.0562 (9)	0.0490 (7)	0.0348 (7)	0.0022 (6)	0.0006 (6)	−0.0041 (5)
O2	0.0736 (8)	0.0566 (6)	0.0648 (7)	0.0189 (5)	0.0331 (6)	0.0119 (5)
C14	0.0581 (8)	0.0468 (7)	0.0336 (6)	−0.0044 (6)	0.0090 (6)	−0.0039 (5)
C11	0.0491 (8)	0.0547 (8)	0.0367 (6)	0.0003 (6)	0.0087 (5)	−0.0003 (5)
C1	0.0497 (8)	0.0413 (7)	0.0470 (7)	0.0024 (6)	0.0078 (6)	0.0017 (5)
C9	0.0444 (7)	0.0478 (7)	0.0452 (7)	0.0016 (5)	0.0109 (6)	0.0023 (5)
C15	0.0495 (7)	0.0492 (7)	0.0409 (7)	−0.0006 (6)	0.0112 (6)	−0.0008 (5)
C10	0.0459 (7)	0.0467 (7)	0.0382 (6)	−0.0028 (6)	0.0057 (5)	−0.0002 (5)
C5	0.0577 (8)	0.0432 (7)	0.0489 (7)	0.0076 (6)	0.0152 (6)	0.0092 (6)
O4	0.0862 (9)	0.0722 (8)	0.0379 (6)	0.0246 (6)	−0.0021 (5)	−0.0010 (5)
C12	0.0463 (8)	0.0503 (8)	0.0407 (7)	0.0023 (6)	0.0050 (6)	−0.0043 (5)
C4	0.0623 (9)	0.0463 (7)	0.0494 (8)	0.0075 (6)	0.0163 (7)	0.0041 (6)
C2	0.0556 (8)	0.0428 (7)	0.0462 (7)	0.0006 (6)	0.0062 (6)	0.0096 (5)
C8	0.0484 (8)	0.0562 (8)	0.0453 (7)	0.0087 (6)	0.0125 (6)	0.0053 (6)
C17	0.0765 (11)	0.0667 (10)	0.0584 (9)	0.0204 (9)	0.0110 (8)	0.0105 (8)
C16	0.0577 (9)	0.0657 (10)	0.0594 (9)	0.0082 (7)	0.0210 (8)	0.0027 (7)
C18	0.0911 (13)	0.0769 (11)	0.0506 (9)	0.0143 (9)	0.0333 (9)	0.0034 (7)
C19	0.0736 (11)	0.0730 (11)	0.0529 (9)	0.0016 (8)	0.0188 (8)	0.0205 (7)

### Geometric parameters (Å, °)

**Table:**

C6—C1	1.3906 (19)	C9—C10	1.4682 (19)
C6—C5	1.395 (2)	C9—H9	0.93
C6—C7	1.4889 (19)	C15—C10	1.3986 (19)
C3—O1	1.3626 (17)	C15—H15	0.93
C3—C2	1.387 (2)	C5—C4	1.380 (2)
C3—C4	1.3906 (19)	C5—H5	0.93
O5—C12	1.3645 (18)	O4—C17	1.413 (2)
O5—C16	1.4294 (19)	C4—H4	0.93
C7—O2	1.2249 (18)	C2—H2	0.93
C7—C8	1.4771 (19)	C8—H8	0.93
O1—C19	1.4240 (19)	C17—H17A	0.96
O3—C14	1.3699 (16)	C17—H17B	0.96
O3—C18	1.415 (2)	C17—H17C	0.96
C13—O4	1.3685 (17)	C16—H16A	0.96
C13—C14	1.393 (2)	C16—H16B	0.96
C13—C12	1.400 (2)	C16—H16C	0.96
C14—C15	1.393 (2)	C18—H18A	0.96
C11—C12	1.390 (2)	C18—H18B	0.96
C11—C10	1.394 (2)	C18—H18C	0.96
C11—H11	0.93	C19—H19A	0.96
C1—C2	1.384 (2)	C19—H19B	0.96
C1—H1	0.93	C19—H19C	0.96
C9—C8	1.326 (2)		
			
C1—C6—C5	118.15 (13)	O5—C12—C11	123.77 (13)
C1—C6—C7	119.52 (12)	O5—C12—C13	116.13 (12)
C5—C6—C7	122.33 (12)	C11—C12—C13	120.06 (13)
O1—C3—C2	124.72 (12)	C5—C4—C3	119.79 (13)
O1—C3—C4	115.06 (13)	C5—C4—H4	120.1
C2—C3—C4	120.22 (13)	C3—C4—H4	120.1
C12—O5—C16	117.36 (12)	C1—C2—C3	119.18 (12)
O2—C7—C8	121.82 (14)	C1—C2—H2	120.4
O2—C7—C6	120.82 (12)	C3—C2—H2	120.4
C8—C7—C6	117.33 (12)	C9—C8—C7	123.60 (13)
C3—O1—C19	118.02 (13)	C9—C8—H8	118.2
C14—O3—C18	117.77 (12)	C7—C8—H8	118.2
O4—C13—C14	118.37 (13)	O4—C17—H17A	109.5
O4—C13—C12	121.95 (14)	O4—C17—H17B	109.5
C14—C13—C12	119.36 (12)	H17A—C17—H17B	109.5
O3—C14—C15	124.40 (13)	O4—C17—H17C	109.5
O3—C14—C13	115.01 (12)	H17A—C17—H17C	109.5
C15—C14—C13	120.59 (13)	H17B—C17—H17C	109.5
C12—C11—C10	120.51 (12)	O5—C16—H16A	109.5
C12—C11—H11	119.7	O5—C16—H16B	109.5
C10—C11—H11	119.7	H16A—C16—H16B	109.5
C2—C1—C6	121.57 (13)	O5—C16—H16C	109.5
C2—C1—H1	119.2	H16A—C16—H16C	109.5
C6—C1—H1	119.2	H16B—C16—H16C	109.5
C8—C9—C10	125.52 (13)	O3—C18—H18A	109.5
C8—C9—H9	117.2	O3—C18—H18B	109.5
C10—C9—H9	117.2	H18A—C18—H18B	109.5
C14—C15—C10	119.90 (13)	O3—C18—H18C	109.5
C14—C15—H15	120	H18A—C18—H18C	109.5
C10—C15—H15	120	H18B—C18—H18C	109.5
C11—C10—C15	119.49 (12)	O1—C19—H19A	109.5
C11—C10—C9	121.44 (12)	O1—C19—H19B	109.5
C15—C10—C9	119.06 (13)	H19A—C19—H19B	109.5
C4—C5—C6	120.99 (12)	O1—C19—H19C	109.5
C4—C5—H5	119.5	H19A—C19—H19C	109.5
C6—C5—H5	119.5	H19B—C19—H19C	109.5
C13—O4—C17	118.42 (12)		
			
C1—C6—C7—O2	−21.4 (2)	C1—C6—C5—C4	−1.2 (2)
C5—C6—C7—O2	158.90 (15)	C7—C6—C5—C4	178.5 (2)
C1—C6—C7—C8	156.7 (2)	C14—C13—O4—C17	−120.1 (2)
C5—C6—C7—C8	−23.0 (2)	C12—C13—O4—C17	66.4 (2)
C2—C3—O1—C19	−5.5 (2)	C16—O5—C12—C11	−3.9 (2)
C4—C3—O1—C19	174.5 (2)	C16—O5—C12—C13	173.9 (2)
C18—O3—C14—C15	−9.2 (2)	C10—C11—C12—O5	175.0 (2)
C18—O3—C14—C13	171.6 (2)	C10—C11—C12—C13	−2.6 (2)
O4—C13—C14—O3	7.7 (2)	O4—C13—C12—O5	−4.4 (2)
C12—C13—C14—O3	−178.7 (2)	C14—C13—C12—O5	−177.7 (2)
O4—C13—C14—C15	−171.4 (2)	O4—C13—C12—C11	173.5 (2)
C12—C13—C14—C15	2.1 (2)	C14—C13—C12—C11	0.1 (2)
C5—C6—C1—C2	3.0 (2)	C6—C5—C4—C3	−1.7 (2)
C7—C6—C1—C2	−176.6 (2)	O1—C3—C4—C5	−177.3 (2)
O3—C14—C15—C10	179.0 (2)	C2—C3—C4—C5	2.8 (2)
C13—C14—C15—C10	−1.9 (2)	C6—C1—C2—C3	−2.0 (2)
C12—C11—C10—C15	2.9 (2)	O1—C3—C2—C1	179.1 (2)
C12—C11—C10—C9	−176.0 (2)	C4—C3—C2—C1	−1.0 (2)
C14—C15—C10—C11	−0.6 (2)	C10—C9—C8—C7	177.7 (2)
C14—C15—C10—C9	178.3 (2)	O2—C7—C8—C9	−12.8 (2)
C8—C9—C10—C11	−4.4 (2)	C6—C7—C8—C9	169.1 (2)
C8—C9—C10—C15	176.7 (2)		

### Hydrogen-bond geometry (Å, °)

**Table:**

*D*—H···*A*	*D*—H	H···*A*	*D*···*A*	*D*—H···*A*
C9—H9···O2^i^	0.93	2.51	3.415 (2)	165
C16—H16A···O2^ii^	0.96	2.55	3.484 (2)	165
C18—H18C···O1^iii^	0.96	2.53	3.332 (2)	142

Symmetry codes: (i) ; (ii) ; (iii) *x*, *y*, *z*−1.
